# Hydrogels Derivatized With Cationic Moieties or Functional Peptides as Efficient Supports for Neural Stem Cells

**DOI:** 10.3389/fnins.2020.00475

**Published:** 2020-05-20

**Authors:** Kristin Glotzbach, Nils Stamm, Ralf Weberskirch, Andreas Faissner

**Affiliations:** ^1^Department of Cell Morphology and Molecular Neurobiology, Ruhr University Bochum, Bochum, Germany; ^2^Faculty of Chemistry and Chemical Biology, TU Dortmund University, Dortmund, Germany

**Keywords:** cationic moieties, extracellular matrix, functional peptides, hydrogels, integrin, neural stem cells, RGD

## Abstract

The increasing incidence of neurodegenerative diseases such as Alzheimer’s or Parkinson’s disease represents a significant burden for patients and national health systems. The conditions are primarily caused by the death of neurons and other neural cell types. One important aim of current stem cell research is to find a way to replace the lost cells. In this perspective, neural stem cells (NSCs) have been considered as a promising tool in the field of regenerative medicine. The behavior of NSCs is modulated by environmental influences, for example hormones, growth factors, cytokines, and extracellular matrix molecules or biomechanics. These factors can be studied by using well-defined hydrogels, which are polymeric networks of synthetic or natural origin with the ability to swell in water. These gels can be modified with a variety of molecules and optimized with regard to their mechanical properties to mimic the natural extracellular environment. In particular modifications applying distinct units such as functional domains and peptides can modulate the development of NSCs with regard to proliferation, differentiation and migration. One well-known peptide sequence that affects the behavior of NSCs is the integrin recognition sequence RGD that has originally been derived from fibronectin. In the present review we provide an overview concerning the applications of modified hydrogels with an emphasis on synthetic hydrogels based on poly(acrylamides), as modified with either cationic moieties or the peptide sequence RGD. This knowledge might be used in tissue engineering and regenerative medicine for the therapy of spinal cord injuries, neurodegenerative diseases and traumata.

## Introduction

The loss of neural cells is followed by a dramatic dysfunction of the central nervous system with symptoms like memory loss, paralysis and ataxia. The various reasons for the loss of neural cells may be based in neurodegenerative diseases like Alzheimer’s disease, amyotrophic lateral sclerosis and multiple sclerosis (Selkoe and Lansbury, 1999; Gironi et al., 2016; Grossman, 2019), as well as traumatic brain injuries or stroke. However, the mammalian brain has a limited regenerative capacity and is not able to cure the damage by replacing lost cells (Gage and Temple, 2013). Symptomatic pharmaceutical treatment for neurodegenerative diseases are available, but so far no effective causal treatments to cure the patients are available (Pandit and Murthy, 2011; Yacila and Sari, 2014; Folch et al., 2016). Therefore, there is a great interest in the research of new methods using stem cells in the field of regenerative medicine. One objective focuses on the opportunities to activate the self-renewal and differentiation abilities of intrinsic neural stem cells (NSCs) of the adult brain. Alternative strategies explore options to implant NSCs or progenitors derived from embryonic central nervous system (CNS) tissues or induced human pluripotent stem cells (hIPSCs) into the damaged CNS of diseased recipients. In this perspective, the work with stem cells constitutes a promising tool in the attempt to treat neurodegenerative diseases (Mahla, 2016; Napoli et al., 2018; Zhou et al., 2019). Not only NSCs, but also mesenchymal and embryonic stem cells can be transdifferentiated or differentiated into neural cell types, e.g., neurons, astrocytes, and oligodendrocytes (Dhara and Stice, 2008; Jang et al., 2010; Selvaraj et al., 2012; Yan et al., 2013; Zemel’ko et al., 2013; Urrutia et al., 2019). One critical factor for the modulation of the behavior of NSCs of the CNS is the extracellular matrix (ECM; Gattazzo et al., 2014; Roll and Faissner, 2014; Faissner and Reinhard, 2015; Ahmed and ffrench-Constant, 2016). The ECM is composed of glycoproteins and proteoglycans that can be divided into heparan sulfate and chondroitin sulfate proteoglycans according to specific core proteins and attached glycosaminoglycan chains. About 300 distinct genes have been attributed to the core matrisome (Naba et al., 2016). The composition of the secreted macromolecules, which build a surrounding scaffold, influences the behavior and maturation of NSCs, as well as the adhesion and migration toward specific brain regions through the interaction of ECM molecules with cell surface receptors. These receptors activate intracellular signaling pathways to modulate the cytoskeleton, activate kinases or induce gene expression (Saghatelyan et al., 2004; Franco and Müller, 2011). Initially, numerous studies examined the ECM as modulating agents in two-dimensional (2-D) *in vitro* cell culture systems. Recently, the notion emerged that the three-dimensional (3-D) organization of the ECM exerts specific effects (Duval et al., 2017; Seidlits et al., 2019). In this perspective, a novel aim consisted of finding an appropriate 3-D scaffold for cultivating cells in what is considered a more natural environment. To this end the natural-derived and artificial hydrogels were developed. These polymers are designed to mimic the *in vivo* characteristics of the ECM, which renders them attractive biomaterials in regenerative engineering (Tibbitt and Anseth, 2009; Geckil et al., 2010; Hellmund and Koksch, 2019; Mantha et al., 2019). The combination of both particular ECM molecules and hydrogels represents a promising tool to regulate the differentiation of stem cells into specific cell types and can not only be used for *in vitro* culture systems, but also in regenerative medicine as implant in injured or diseased brains (Guan et al., 2017; Kim and Cho, 2018). In this mini review we intend to give an overview about the influence of the ECM on the development of NSCs, particularly in the context of modified hydrogels and their applicability in regenerative medicine.

## Neural Stem Cell Fate Depends on Extracellular Matrix Composition

In the developing and adult CNS stem cells are located in so called stem cell niches. The stem cells and their descendants in these special compartments are surrounded by supporting cells, proximal blood vessels and a special composition of ECM molecules, which are called fractones (Kazanis and ffrench-Constant, 2011; Rojas-Ríos and González-Reyes, 2014; Theocharidis et al., 2014). The ECM environment comprises different glycoproteins, like tenascins and laminins, and proteoglycans, such as chondroitin or heparan sulfate proteoglycans, which have a major impact on the maintenance and development of NSCs (Faissner and Reinhard, 2015). Especially the expression pattern of the glycoprotein tenascin-C makes it an attractive molecule for neural stem cell research. It was found expressed in the developing brain, more precisely in the stem cell regions (Gates et al., 1995; Steindler et al., 1996; Fietz et al., 2012), as well as after injuries and in tumors (Roll and Faissner, 2019). Tenascin-C is a hexameric glycoprotein, whereby one monomer consists of EGF-like repeats, eight constant and six alternatively spliced fibronectin III domains in mice, resulting in a variety of isoforms. In the developing cerebellum 24 different variants of tenascin-C were found (Joester and Faissner, 1999, 2001; Theocharidis and Faissner, 2012), whereas neurospheres derived from NSCs express 20 isoforms (von Holst et al., 2007). Tenascin-C was found to interact with a diversity of ECM molecules, receptors and growth factors, which activate different signaling cascades. This indicates a great spectrum of functions based on the number of isoforms and the different cell types. Thus it can have repulsive, inhibitory or stimulatory effect on axon growth and guidance (Faissner, 1997; Joester and Faissner, 2001; Rigato et al., 2002; Michele and Faissner, 2009), as well as on cell migration, cell attachment, and cell spreading and cell survival (Giblin and Midwood, 2014). Other glycoproteins, which are prominent for the neural stem cell niche, are laminins (Mercier et al., 2002; Kerever et al., 2007). They are heterotrimeric molecules and are a major component of the basement membrane (Colognato and Yurchenco, 2000). They interact with a variety of molecules, like other matrix molecules, and cell surface receptors. Via the interplay with receptors, laminins may influence the behavior of the cells through the activation of intracellular signaling pathways and thus is responsible for differentiation, survival, and movement and maintenance of the cells (Colognato and Yurchenco, 2000). Laminins are important in the developing cortex and its disruption results in cortical disorganization (Halfter et al., 2002; Radner et al., 2013). The importance of laminin for NSCs can also be seen *in vitro*, where laminin is the preferred substrate of adult NSCs (Pollard et al., 2006), and promotes the proliferation of human NSCs (Hall et al., 2008). On the other hand, laminin is also important for the maturation of neural cells, for example for the differentiation of neural cells into neurons and astrocytes (Flanagan et al., 2006). In particular, it is a well-known neurite outgrowth inducing and neuron differentiation supporting substrate (Plantman et al., 2008). Another important protein of the ECM is fibronectin. It is expressed in the developing cortex and the subependymal niche, the location of adult NSCs (Letourneau et al., 1994; Morante-Redolat and Porlan, 2019). It is a dimer and has different isoforms, that arise from alternative splicing (ffrench-Constant, 1995), and therefore plays an important role in cell adhesion, migration and differentiation (Mosher, 2012; Carsons, 2018). Additionally it could be shown to promote neurite outgrowth (Tonge et al., 2012) and plays a major role in regeneration of peripheral nerves (Lefcort et al., 1992).

**FIGURE 1:**
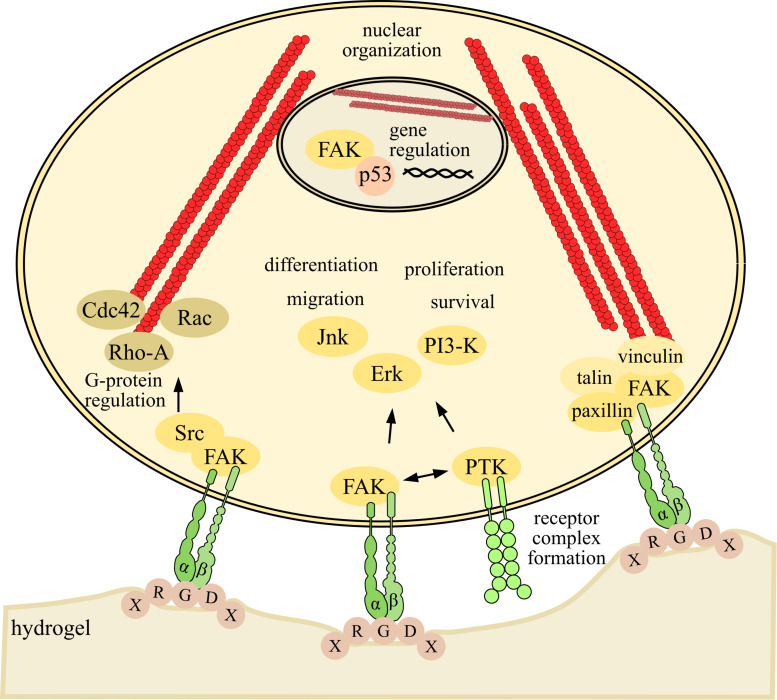
Integrin-mediated intracellular signaling activated by adhesion of cells to a RGD-modified hydrogel. The crosstalk of integrin to the peptide sequence RGD activates many signaling pathways, which are mostly initiated by the activation of the focal adhesion kinase (FAK). Together with the protein tyrosine kinase Src, FAK may regulate small GTPases of the RhoA subfamily like CDC42, Rac and Rho-A, which may modulate the actin cytoskeleton. Furthermore the cytoskeleton may be regulated by the activation of the focal adhesion core region, which is build by FAK and paxillin in the first stratum, followed by talin and vinculin, which interact with actin through molecules like α-actinin. The interaction with actin has influences on the adhesion and migration of cells, as well as on the gene expression via the organization of the nucleus, which is linked to the cytoskeleton. Additionally integrins may have direct influence on the gene expression through the shuttle of FAK into the nucleus. There FAK targets the ubiquitination of the cell cycle mediator p53 and may act as a transcription co-regulator. The differentiation, proliferation and survival of the cells may be influenced through integrins alone or in combination with growth factor receptors like the epidermal growth factor receptor and the platelet-derived growth factor via the activation of mitogen-activated protein kinase/Erk, Jnk or phosphoinositide 3-kinase/Akt (Schwartz and Ginsberg, 2002; Yamada and Even-Ram, 2002; Giancotti and Tarone, 2003; Guarda et al., 2009; Ivaska and Heino, 2011; Dalby et al., 2014).

The major receptor family for ECM molecules are the integrins, which are present in the human CNS in 23 different variants depending on their combination of their α- and β-subunit (Gardiner, 2011; Berezin et al., 2014; Senkov et al., 2014). Particularly the β_1_-integrin subunit is strongly represented in neural progenitor cells (NPCs), for example in neurospheres and inside the ventricular zone of the developing cortex, which is the location of the proliferating NSCs (Campos et al., 2004; Hall et al., 2006). It is important for neural stem cell maintenance and its inactivation or deletion caused process retraction and altered neurogenesis (Graus-Porta et al., 2001; Loulier et al., 2009; Radakovits et al., 2009; Fietz et al., 2010). Furthermore, integrins cooperate with growth factor receptors and affect the activation of several intracellular signaling pathways, like Erk and phosphoinositide 3-kinase through the activation of protein tyrosine kinases like the focal adhesion kinase (FAK) and Src, as well as G-proteins like Cdc42, Rac and Rho, which influences the behavior of cells especially via the modulation of actin (see Figure 1; Dalby et al., 2014). The decision which pathway will be activated depends on different parameters, for example the stiffness of the environment. So it could be shown that the phosphoinositide 3-kinase and the Rho-associated–protein-kinase were two parallel signaling pathways, which were activated by the same input of the ECM to integrin, but have opposite effects regarding their protrusive activity. The outcome of the activation of this signaling depends on the balance between these two pathways, which is associated with the stiffness of the ECM environment (Park et al., 2018). These results support the conclusion that the interaction of ECM molecules with integrin receptors has an influence on the maintenance and development of NSCs (Campos et al., 2006; Alam et al., 2007; Rallis et al., 2010; Arora et al., 2012; Brizzi et al., 2012).

Remarkably, the receptor binding sites of the ECM glycoproteins can be attributed to short amino acid sequences such as Arg–Gly–Asp (RGD; Ruoslahti and Pierschbacher, 1987). This peptide was originally discovered in fibronectin but is also encoded in several other ECM molecules like vitronectin, collagens and fibrinogen. Along these lines, functionally active peptide sequences uncovered in other ECM molecules include IKVAV from laminin-1 and VSWRAPTA, and VFDNFVLK from tenascin-C glycoproteins (Tashiro et al., 1989; Mercado et al., 2004; Jarocki et al., 2018). These peptides are all known to support the attachment of cells and/or to promote the outgrowth of neurites. The discovery of these short functional peptides opened new doors for the researchers in two different ways. On the one hand the handling of short peptides compared to larger protein domains is more convenient. Especially the tethering of these sequences to substrates and materials by chemical coupling is more straightforward than the use of large glycoproteins. On the other hand, however, the small peptides are limited in their functional potential in contrast to the integral proteins they originate from, which may have more than one biologically active domain, and hence offer more receptor binding sites to interacting cellular partners. In view of the fact that the peptides target one distinct receptor they may be more specific for the regulation of the behavior of NSCs and represent a promising tool in regenerative medicine.

## Hydrogels as Matrices for Cell Cultivation

Extracellular matrix molecules are often used in cell culture systems as coatings to create a natural cellular environment. However, it was found that the cells not only need the cell-cell- and cell-matrix-interaction, but also the mechanical support to adopt the behavior of cells inside the organism (Lopes et al., 2019). One modern approach for the cultivation of stem cells is the use of hydrogels as scaffolds with the ability to mimic the ECM of those cells. Hydrogels are three-dimensional hydrophilic polymeric networks that are insoluble in water due to their crosslinks which can be formed by covalent but also non-covalent bonds like hydrogen bonds or ionic bridges (Kloxin et al., 2009). Nowadays many different polymers are used as hydrogels such as natural derived polymers like Collagen (Trappmann et al., 2012), Hyaluronic acid (HA; Collins and Birkinshaw, 2013), Chitosan (Payne and Raghavan, 2007), or Gelatin (Nichol et al., 2010). But also different fully synthetic polymers like poly(ethylene glycol) (Slaughter et al., 2009), poly(hydroxyethyl methacrylate) (Guiseppi-Elie, 2010), or more complex structures like block copolymers (Tokarev and Minko, 2009) are being tested. While natural polymers often have very good biocompatibilities and mechanical properties, they are also very expensive in their production, are often not very well characterized and display considerable batch-to-batch variation. On the other hand, fully synthetic hydrogels are cheap in their production and properties such as their mechanical stability, hydrophilicity, and biocompatibility can be easily adjusted to the needs of different cell types. Additionally, these gels can be modified with different moieties to influence the behavior of cells during the cultivation process, which is necessary if the cells are to be used to treat different diseases or disabilities. The most commonly used moieties which are known to influence the behavior of cells are cationic charges or peptide sequences like the very well-known Arg-Gly-Asp (RGD) sequence derived from fibronectin or laminin α1 and α5 chain (Tashiro et al., 1991; Sasaki and Timpl, 2001), Ile-Lys-Val-Ala-Val (IKVAV) derived from laminin-1 or Val-Phe-Asp-Asn-Phe-Val-Leu-Lys (VFDNFVLK) derived from tenascin-C (Zhu, 2010).

As early as in 1974 Yavin and Yavin demonstrated that the polycationic polymer polylysine enhanced the adhesion of cells to the surface of petri dishes through ionic interactions of the positively charged amine side chain of the lysine and the negatively charged cell membrane (Yavin and Yavin, 1974). It was shown that 75% of brain cells readily adhered to the surface in only 15 minutes and afterwards proliferate and differentiate. Comparing the results of polylysine to different other amino acid coatings containing thiols or carboxylic acids, they could attribute the enhanced adhesion of the cells to the present free amine group of lysine. Several years later Cai et al. (2012) reported a hydrogel system comprised of poly(ethylene glycol) diacrylate and poly(L-lysine) or [2-(methacryloyloxy)ethyl]-trimethylammonium chloride, which enhances proliferation, differentiation, and survival of encapsulated NPCs (Cai et al., 2012). It was demonstrated that compared to a neutral reference the viability of cells on hydrogels bearing cationic charges was significantly higher after day 1 and day 7. Furthermore, the proliferation on these gels was also much faster and an enhanced differentiation could be observed in both gels bearing cationic charges. Some differences between the two gels were visible which indicated a difference of the cationic moieties in lysine bearing a primary amine group and in [2-(methacryloyloxy)ethyl]-trimethylammonium chloride gels bearing a quaternary ammonium group (Cai et al., 2012). The differentiation was also dependent on the stiffness of the hydrogel in that softer gels that are closer to natural brain tissue were favored. It turned out that poly(L-lysine) is superior to gels bearing quaternary ammonium ions and is therefore a promising material for the regeneration of the CNS (Cai et al., 2012). Hynes et al. (2009) reported that lateral concentrations of amines ranging from 0.1 to 3.0 μmol mg^–1^ are sufficient to promote the survival and proliferation of NSCs on hydrogels without further modifications (Hynes et al., 2009). The best results regarding migration and formation of neurons were obtained from gels with an elastic modulus of 3800 to 5300 Pa and a lateral concentration of 0.32–0.60 μmol mg^–1^. While these results already show that hydrogels containing cationic charges are promising tools to influence the behavior of NSCs, many researchers focus their interest on hydrogels with peptide sequences derived from natural proteins from the ECM (Sun et al., 2017; Stukel and Willits, 2018).

One of the most intensively investigated peptide sequences for cell cultivation is the RGD sequence. Saha et al. (2007) established a fully synthetic hydrogel network with different layers, a so called interpenetrating polymer network, with poly(ethylene glycol), and poly(acrylamide) as their main component and an RGD functionalized upper layer (Saha et al., 2007). With this hydrogel system they could demonstrate that a lateral peptide concentration greater than 5.3 pmol/cm^2^ on the surface was sufficient to support adhesion of NSCs to the gel via integrin receptors. It was shown that the RGD sequence used in this study provided comparable results as the glycoprotein laminin-1 with respect to cell adhesion, differentiation and morphology. Also, a concentration dependent increase in cell proliferation could be shown. In contrast to these observations cells did not adhere on gels containing the peptide IKVAV that is derived from laminin-1 and no difference in cell differentiation or proliferation was observed. This indicates that RGD alone can substitute laminin-1 with regard to some aspects of cell differentiation. Furthermore, the data emphasizes that the different peptide sequences contained in ECM glycoproteins have different functions and effects on neural stem cell behavior. In a subsequent paper Saha et al. (2008) further described the importance of the substrate modulus on the behavior of NSCs (Saha et al., 2008). They studied the influence of the substrate modulus in a range of 10 Pa to 10 kPa with the same hydrogel system mentioned before. The adult NSCs proliferated and differentiated on all gels with a modulus greater than 100 Pa and a peak level of the neuronal marker β-III tubulin was observed on gels with a modulus of around 500 Pa which resembles the stiffness of brain tissue. Also the neurite outgrowth was increased in hydrogels with a similar stiffness of 400 Pa in a HA based RGD-modified hydrogel (Tarus et al., 2016) and in a PEGDA-RGD-based hydrogels the neurite outgrowth was increased at a stiffness of 0.1 and 0.8 kPa, which were the lowest consistence tested (Stukel and Willits, 2018). Together this indicates that neurites preferentially grow toward an environment that is less stiff (Long and Huttner, 2019). Additionally Stukel and Willits (2018) carried out adhesion studies with the 2-D modified PEGDA hydrogels and could show that the concentration of the peptide and the cell density are important for the adhesion effect of RGD. More precisely they exposed that a high concentration of 2.5 mM RGD decreased the adhesion at a cell density of 50 × 10^3^ cells cm^–2^, whereas a concentration of 0.1 and 1 mM, as well as a lower cell density of 10 × 10^3^ cm^–2^, exhibited a better result. Another 3-D artificial hydrogel based on PEG was modified with RGD in combination with tenascin-C (Naghdi et al., 2016). This hydrogel promoted the viability of NSCs and provoked an increased differentiation of NSCs into a neuronal phenotype (Table 1).

**TABLE 1:** Different modified hydrogels with their effect on NSC.

Hydrogel	Moiety	Effect	References
RADA16-I self-assembling peptides 2D and 3-D	RGD	Increased proliferation and differentiation of NSC	Cunha et al., 2011
RADA 16 self-assembling peptides	IKVAV	Increased proliferation, migration and differentiations into neurons of NSC	Zhang et al., 2010
RADA16 self-assembly peptides, RADA16 cyclo-RGD 3-D	RGD	Increased proliferation and neuronal differentiation of NSC	Gao et al., 2017
Polyethylene glycol–hydrogel 3-D	RGD and tenascin-C	Higher viability, proliferation, and differentiation into neuronal phenotype and neurite outgrowth of bone marrow stem cells-derived neurospheres	Naghdi et al., 2016
PEGDA 2D	RGD	NSPC adhesion and neurite extension	Stukel and Willits, 2018
Hyaluronic acid matrix 3-D	IKVAV and LRE (laminin)	Neurite extension of embryonic stem cells through matrix metalloprotease-dependent mechanism	Perera et al., 2019
Hyaluronic acid hydrogel 3-D	RGD	Neurite outgrowth of neural progenitor cells	Tarus et al., 2016
Hyaluronic acid-based hydrogels 3-D	RGD	Improved viability and proliferation of NSCs	Seidlits et al., 2019
Hyaluronic acid-based hydrogel 3-D	RGD and heparin	Increased survival of midbrain dopamineric neurons after implantation	Adil et al., 2017
Gellan gum	GRGDS	Pronounced differentiation, proliferation, migration and expansion throughout the hydrogel, of NSCs	Silva et al., 2012
Poly (methylsulfone acrylate-*co*-acrylamide-*co*-acrylic acid-*co*-N,N’-methylene-bis-acrylamide)	IKVAV	Differentiation of NSCs into neurons	Farrukh et al., 2017
Poly (2-hydroxyethyl methacrylate-*co*-2-aminoethyl methacrylate-*co*-ethylene dimethacrylate)	RGDS and SIKVAVS	RGDS shows better cell attachment, proliferation, and growth than SIKVAVS	Macková et al., 2016
Polyethylene glycol (PEG), agarose and polyacrylic acid 3-D	RGD	Increased proliferation of NSC and more cells in active phase	Mauri et al., 2018
Elastin-like polypeptides (ELP), 3-D	RGD	Neurite outgrowth at optimal gel stiffness	Lampe et al., 2013
Elastin-like polypeptides (ELP), 3-D	RGD + protease degradation sites	Matrix remodeling is required for NPC stemness maintenance and differentiation	Madl et al., 2017, 2019

**FIGURE 2:**
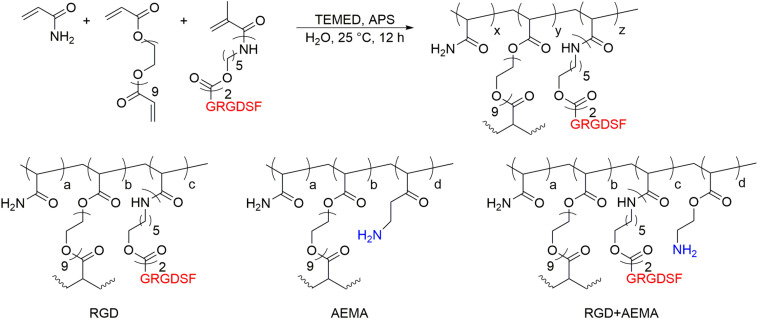
Exemplary synthesis of a synthetic hydrogel system and examples for hydrogels bearing cationic moieties and the RGD sequence.

While cationic charges, substrate modulus and peptides on their own are promising tools to influence the behavior of NSCs, they are very often not combined in a single hydrogel (Jaiswal et al., 2013; Macková et al., 2016). Therefore, Sallouh et al. (2017) investigated the effect of both the cationic charge and the peptide sequence GRGDSF in a cross-linked hydrogel system based on poly(acrylamide). In total the effect of three different hydrogels, namely a neutral gel bearing the peptide GRGDSF, a gel with cationic charges based on 2-aminoethylmethacrylate and a gel bearing both cationic charges and the peptide GRGDSF, on the behavior of NSCs were analyzed (see Figure 2).

The analysis of neural stem cell adhesion showed a 10.8-fold increase in the number of adherent cells for the cationic gel and a 22-fold increase for the gel that combined cationic charges with the RGD peptide compared to the pure RGD-functionalized gel with a RGD-concentration well below the threshold concentration of Saha et al. (2007). These results suggest that early cell adhesion is mediated by the electrostatic interaction of cationic charges with the negatively charged pericellular matrix of the cell membrane. The importance of the pericellular matrix for early cell adhesion has also been reported for other cell types such as osteoblasts, chondrocytes or epithelial cells (Cohen et al., 2003; Fotia et al., 2013; Scrimgeour et al., 2017). RGD-integrin interaction and focal adhesion formation take place in a second step when the cell-substrate distance is in a range of 25–50 nm leading again to a doubling in adherent cells compared to the cationic gel whereas the pure RGD-functionalized gel did not show any cell adhesive properties. The results suggest a synergistic effect of the cationic moieties (non-specific electrostatic interactions with the pericellular matrix) and the RGD-peptides (specific interactions with integrins) that take place on a different distance and time scale.

Although the majority of studies using hydrogels investigated cell behavior in two dimensions (2-D) on top of a hydrogel film 3-D cell encapsulation has gained significant interest in the past years since 3-D hydrogel constructs may mimic the tissue environment of the cells more accurately (Caliari and Burdick, 2016). Moreover, 3-D cell encapsulation has become an important tool for cell delivery and therapy based on 3-D bioprinting or injectable hydrogels (Thomas and Willerth, 2017; Liang et al., 2019).

One hydrogel system that has been used for 3-D applications is based on self-assembling peptides (SAP). The components of these hydrogels, for example the RADA16 peptide, may also be modified with peptide sequences like RGD or IKVAV (Table 1; Zhang et al., 2010; Cunha et al., 2011; Gao et al., 2017). It could be shown that the cells cultured in RGD-functionalized SAP proliferate and differentiate, for example into neurons, and more than in unmodified hydrogels (Cunha et al., 2011; Gao et al., 2017). The modification with IKVAV promotes beside the proliferation of NSCs also the migration of the NSCs into the SAP hydrogel to form a 3-D culture in comparison to defunctionalized SAPs (Zhang et al., 2010). Furthermore, the differentiation into neurons was promoted by IKVAV-modified SAPs, whereas the differentiation into astrocytes was decreased.

An often used hydrogel system are the HA based hydrogels (Table 1; Tarus et al., 2016; Adil et al., 2017; Perera et al., 2019; Seidlits et al., 2019). The HA based 3-D hydrogel alone shows an increased differentiation of human NSCs into oligodendrocytes and neurons and a decreased formation of reactive astrocytes compared to a 2-D laminin-coated culture (Seidlits et al., 2019). Additionally, the modification with RGD increased the viability of the human NSCs. Furthermore the modification with RGD exhibits the neurite outgrowth into the soft hydrogel of NPC (Tarus et al., 2016; Adil et al., 2017). Moreover 3-D HA based hydrogels, which were modified with RGD and heparin, were used in transplantation studies (Adil et al., 2017). These hydrogels support the neuronal differentiation of midbrain dopaminergic neuronal progenitors and enhance the survival of implanted midbrain dopaminergic neurons after injection into the adult striatum of rats. Another class of bioactive materials for cell encapsulation is based on elastin-like polypeptides (ELPs; Lampe et al., 2013). These materials were fabricated by protein engineering and studied the effect of RGD-concentration and biomechanics on neurite outgrowth from dorsal root ganglia. Greatest neurite outgrowth was found for ELP gels with elastic moduli between 0.5 and 2.1 kPa and identical RGD-concentrations. In a more recent study, they could show that the stemness of a NPC culture within a 3-D ELP gel modified with RGD-peptides and protease degradation sites is correlated with gel degradability and showed that matrix remodeling is required for NPC stemness maintenance in 3-D gels (Madl et al., 2017). This matrix remodeling was also necessary to enable NPC differentiation into astrocytes and neurons (Madl et al., 2019). This study shows the importance of 3-D hydrogels for regenerative medicine.

While some hydrogels are already used for medical applications such as wound treatments, many challenges have to be overcome for them to be used in patients for different applications. Using human embryonal stem cells for research is associated with ethical problems. In this respect, adult stem cells and induced pluripotent stem cells circumvent these problems and are the main target in clinical trials for hydrogels. Using patient-derived cells reduces immune responses of the body to the hydrogel implant. Furthermore, endogenous stem cells inside the injured tissue may be stimulated by the implanted modified hydrogel to proliferate and differentiate into the required cell types (Liu et al., 2020). On the other hand, the patient-derived cells may be burdened by the same neuropathological deficits that cause the neurodegenerative disease. Another major challenge is the vascularization of the newly built tissue, which is important to provide transportation of nutrients. Mainly two different approaches are used at the moment to overcome this latter challenge. The first one is a cell-based strategy which uses endothelial cells to induce neo-angiogenesis and the second one is a scaffold-based strategy which focuses on providing vessel like structures in the scaffold (Novosel et al., 2011). Other issues refer to the ease of handling the hydrogel during the application and the cost efficiency. Depending on the cells the scaffolds need different mechanical properties, but surgeons need a durable and easy to handle implant for the application. One solution might be the injection of fluid gels with subsequent *in situ-*gelation inside the injury (Yang et al., 2014). Depending on the injury or disease type the hydrogels have to be stable or degradable (Li et al., 2012; Lim et al., 2018). The degradability is particularly important for the invasion and the repopulation of the hydrogel by invading cells. Therefore, the hydrogel has to be modified with biodegradable groups like esters that can be broken by enzymes secreted by the cells of interest, for example matrix metalloproteases. Another problem of most hydrogels is the orientation of the structure inside the hydrogel, because many tissues have an ordered structure and provide cells a direction to grow and migrate (Rose et al., 2020). In addition, natural and synthetic hydrogels are often subject to numerous modification steps and therefore not very cost efficient. Overcoming these challenges may eventually lead to competence to generate fully functional implants, or even full organs using hydrogels as a matrix.

In summary, the great interest in regenerative medicine to cure neurodegenerative diseases resulted in an expansion on research concerning the development of the nervous system. Soon it became obvious that ECM molecules, like laminins, tenascins, and fibronectins, are important for normal brain development. Single domains and peptides of these molecules have specific functions regarding the behavior of NSCs by activating intracellular signaling pathways via binding to cell surface receptors, like integrins. Nevertheless, *in vitro* experiments using distinct ECM molecules generate impressive results, but are limited in their abilty to mirror the *in vivo* situation in an organism. In order to create a more natural environment artificial and natural-derived hydrogels are investigated. Recent advances in materials science have led to innovative biomaterials for use in stem cell research. While in the beginning the research effort was mainly focused on a better understanding of the influencing parameters such as topological cues, biomechanics or bioactive components on neural stem cell behavior in 2-D hydrogel films, there is a clear shift toward the development and application of 3-D cell encapsulation materials that resemble the natural environment of NSCs more closely. Synthetic 3-D hydrogels with multiple functionalities such as peptides or growth factors have been demonstrated to support very efficiently the differentiation of human NSCs into neurons and the formation of neural networks (Marchini et al., 2019). Therefore, the development of synthetic or natural materials that are able to mimic important features of the ECM and can be utilized for 2-D and 3-D cell experiments remains a topic of high priority in stem cell research.

## Author Contributions

KG, NS, RW, and AF wrote and revised the manuscript. KG and NS designed the figures.

## Conflict of Interest

The authors declare that the research was conducted in the absence of any commercial or financial relationships that could be construed as a potential conflict of interest.
